# Characterization of C-terminal structure of MinC and its implication in evolution of bacterial cell division

**DOI:** 10.1038/s41598-017-08213-5

**Published:** 2017-08-08

**Authors:** Shaoyuan Yang, Qingya Shen, Shu Wang, Chen Song, Zhen Lei, Shengnan Han, Xiaoying Zhang, Jimin Zheng, Zongchao Jia

**Affiliations:** 1 College of Chemistry, Beijing Normal University, Beijing, 100875 China; 2 Department of Biomedical and Molecular Sciences, Queen’s University, Kingston, Ontario, K7L 3N6 Canada

## Abstract

Proper cell division at the mid-site of Gram-negative bacteria reflects stringent regulation by the *min* system (MinC, MinD and MinE). Herein we report crystal structure of the C-terminal domain of MinC from *Escherichia coli* (*Ec*MinC_CTD_). The MinC_CTD_ beta helical domain is engaged in a tight homodimer, similar to *Thermotoga maritima* MinC_CTD_ (*Tm*MinC_CTD_). However, both *Ec*MinC_CTD_ and *Tm*MinC_CTD_ lack an α-helix (helix3) at their C-terminal tail, in comparison to *Aquifex aerolicu* MinC_CTD_ (*Aa*MinC_CTD_) which forms an extra interaction interface with MinD. To understand the role of this extra binding element in MinC/MinD interactions, we fused this helix (*Aa*helix3) to the C-terminus of *Ec*MinC and examined its effect on cell morphology and cell growth. Our results revealed that *Aa*helix3 impaired normal cell division *in vivo*. Furthermore, results of a co-pelleting assay and binding free energy calculation suggested that *Aa*helix3 plays an essential role in *Aa*MinCD complex formation, under the circumstance of lacking MinE in *A. aerolicu*. Combining these results with sequence analysis of MinC and MinD in different organisms, we propose an evolutionary relationship to rationalize different mechanisms in cell division positioning in various organisms.

## Introduction

Bacterial cells have a fundamental need to divide by binary fission through accurate spatial and temporal regulation of septum formation, producing two daughter cells of equal size^1^. In the Gram-negative bacterium *E. coli*, a tubulin like protein (FtsZ) polymerizes at mid-cell to form a ring structure (Z-ring) to ensure cell division, which is often spatially regulated by the Min system, including MinC, MinD and MinE, through a pole-to-pole oscillation^2, 3^. MinC, as an inhibitor of the Z-ring, is recruited to the membrane by MinD and prevents FtsZ polymerization. MinE has been shown as an anti-MinCD component. The binding of a MinE dimer to MinCD has been reported to trigger the release of MinC followed by stimulating the ATPase activity of MinD and its dissociation from the membrane, thereby protecting the central division site to avoid inhibition by MinCD^4^. MinC contains two separate domains: an N-terminal domain, which directly interacts with FtsZ and prevents its polymerization; and a C-terminal domain, which forms a constitutive dimer and interacts with MinD as well as with FtsZ^5^. Ghosal *et al*. found that, *in vitro*, MinC and MinD together form a new class of nucleotide-dependent and alternating copolymeric filaments^6^. These copolymers were shown not to be physiologically relevant^7^, but are still useful in studying MinD-MinC interactions in a biochemical context. During the oscillation, MinD, along with MinC and MinE, is present in a polar zone flanked near mid-cell by the MinE ring^4, 8, 9^. In this mechanism of cell division positioning, each of the three proteins (MinC, MinD and MinE) plays indispensable functional roles and must be present. In contrast, in the Gram-positive bacterium *B. subtilis*, the MinCD complex does not appear to oscillate because there exists no MinE. Due to interaction with the pole-anchored DivIVA, the MinCD complex remains concentrated in two polar regions, thereby preventing division from taking place near the poles^10, 11^. In this scenario, MinJ is needed to bridge DivIVA and MinD^12, 13^, resulting in a different cell division pattern from *min* oscillation in the Gram-negative bacteria.

Archaea are a third domain of life in addition to Bacteria and Eukarya^14^. Archaea were initially viewed as extremophiles living in harsh environments, such as hot springs and salt lakes^15^. The mechanisms of cell division in Archaea differ from those in bacteria in many aspects, including DNA replication and membrane organization^16^. Almost all members of the Euryarchaeota, one of the five archaeal phyla, encode FtsZ and, thus, are thought to possess a bacterial-type division mechanism^17, 18^. However, the exact mechanism of cell division of Archaea has not been investigated and thus remains completely unknown. Further, the evolutionary relationship with regard to cell division between Archaea and Eukaryotes is also unclear. *A. aeolicu* is one of a handful of species in the *Aquificae* phylum, an unusual group of thermophilic bacteria that is thought to be the oldest species of bacteria^19^. Similarly, *T. maritima* resides in hot springs as well as hydrothermal vents^20^. The ideal environment for the organism is a water temperature of 80 °C (176 °F). Although *T. maritima* and *A. aeolicu* have been determined as members of Gram-negative bacteria, they reside in extreme environments similar to members of the domain Archaea^21^. Therefore, study of *T. maritima* and *A. aeolicu* will help understand the interaction and evolutionary relationship among Bacteria and Archaea.

In this work, we determined the crystal structure of the dimeric C-terminal domain of MinC from *E. coli* (*Ec*MinC_CTD_). *E*cMinC_CTD_ forms a dimer between the two β-sheets in each subunit, as observed in the *Tm*MinC_CTD_ structure^22^. However, both *Ec*MinC_CTD_ and *Tm*MinC_CTD_ lack an α-helix (helix3) at their C-terminal tail compared to *Aa*MinC_CTD_ which forms another interaction interface with MinD^6^. By fusing helix3 to the C-terminus of *Ec*MinC, we studied its effect on cell morphology and cell growth, revealing that *Aa*helix3 impaired normal cell division in *E. coli*. Results of a co-pelleting assay and binding free energy calculation further led to our conclusion that the *Aa*helix3 enhances the interaction of *Aa*MinCD complex, which may hinder the role of MinE in the dissociation of MinCD complex. Sequence analysis shows that there is no *minE* gene and MinC with the extra helix3 in non-oscillation cell division systems. Taken together, we propose an evolutionary relationship to explain the different mechanisms in cell divisions in Bacteria and Archaea.

## Results

### Structural characterization of *Ec*MinC_CTD_

Since full-length *Ec*MinC structure has not been determined, we initially aimed to crystallize the full-length protein. After 3~6 weeks, crystals grew to the maximum size (Fig. S1). SDS-PAGE analysis of the harvested crystals showed that these crystals resulted from a degradation fragment of the MinC protein at a molecular weight of ~13 kD. Furthermore, western blotting results demonstrated that this fragment of MinC had the C-terminal 6 × His tag (data not shown), which suggested that our crystals were from *Ec*MinC’s C-terminal domain (MinC_CTD_). This is likely caused by degradation of the flexible linker between the N- and C-terminal domains of MinC, which is easily susceptible to non-specific proteolysis. Subsequently, we created the C-terminal domain (pET22b-MinC_CTD_) construct for crystallization. However, MinC_CTD_ protein was very unstable and prone to precipitation. As a result, we went back to the initial method of using full-length MinC to obtain MinC_CTD_ crystals. It is inferred that MinC_NTD_, although cleaved off over time, initially helps to promote the correct folding and solubility of MinC_CTD_.

**Figure 1:**
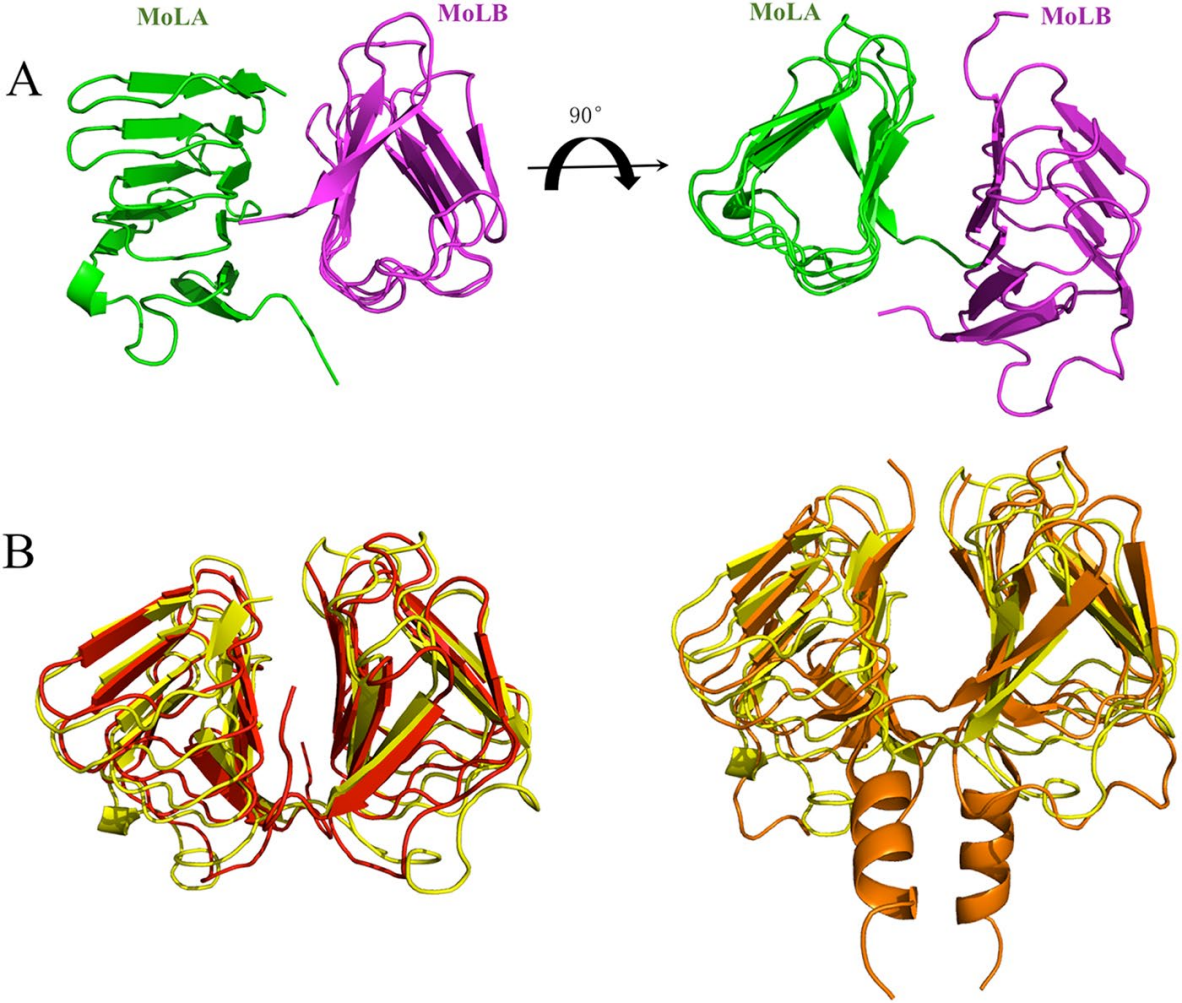
Overall structure of the dimeric MinC C-terminal domain from *E. coli* (*Ec*MinC_CTD_). (**A**) The ribbon diagrams of two monomers are in green and magenta. (**B**) Left: structural comparison of *Ec*MinCCTD (yellow) and the *Tm*MinC C-terminal domain (*Tm*MinCCTD, red). Right: structural comparison of *Ec*MinCCTD (yellow) and the *Aa*MinC C-terminal domain (*Aa*MinCCTD, orange). Superimposition was performed using the program *PyMOL*.

The crystal structure of *Ec*MinC_CTD_ was solved at 3.0 Å resolution using the molecular replacement method. The search model was the C-terminal domain of *Tm*MinC (PDB: 1HF2). *Ec*MinC_CTD_ forms a dimer within the asymmetric unit via domain swapping (Fig. 1A). The tight dimer formed through the MinC_CTD_ beta helical domain is similar to that of *Tm*MinC_CTD_ (Fig. 1B, left). The two MinC_CTD_ structures superimposed with a root-mean-square deviation (RMSD) of 1.4 Å for the 110 Cα atoms (residues 122–231).

**Figure 2:**
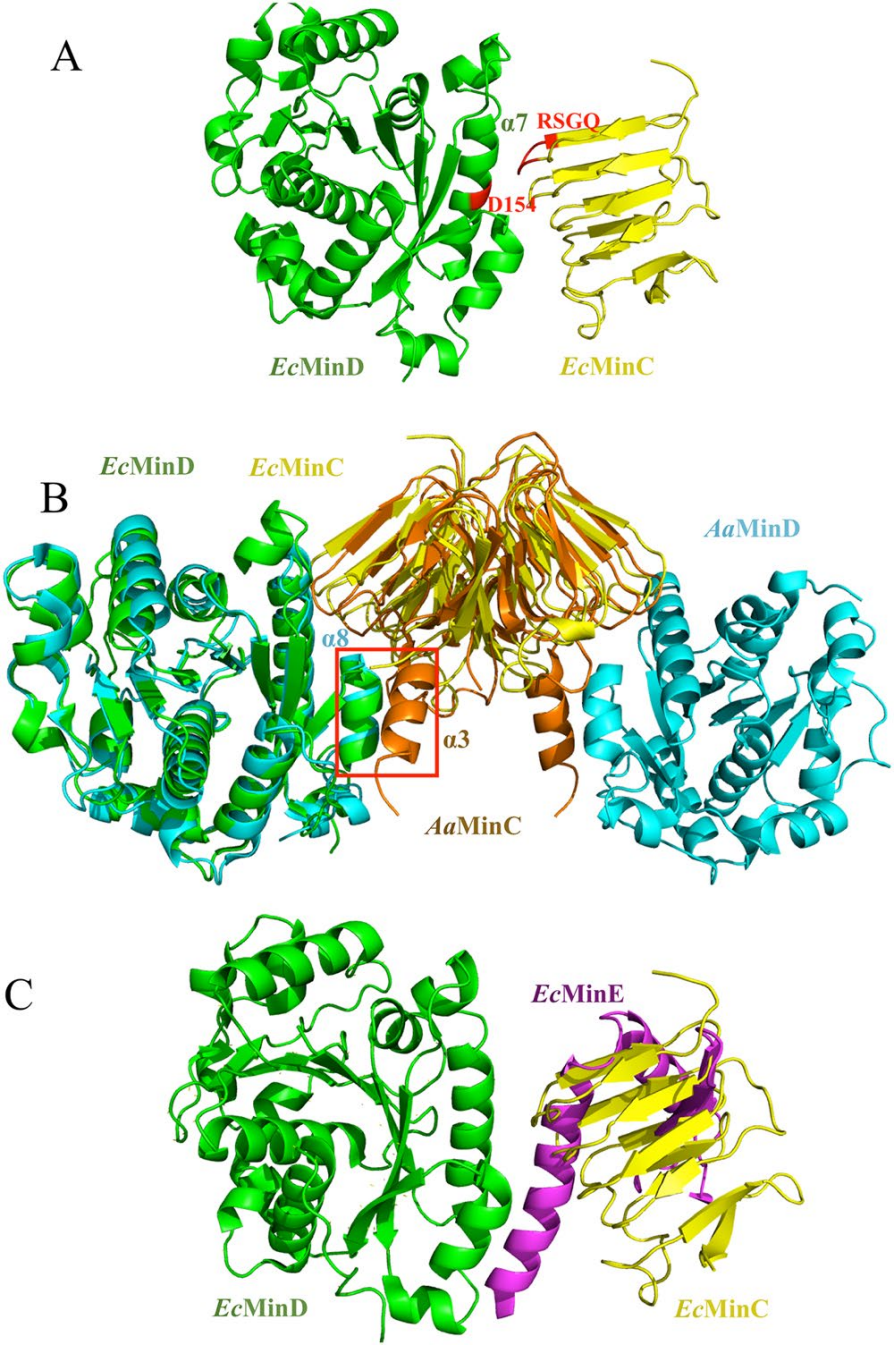
Structure of assembled *Ec*MinCD complex and its superimposition with *Aa*MinCD complex and *Ec*MinDE complex. (**A**) Assembled *Ec*MinCD complex using *Ec*MinC_CTD_ and *Ec*MinD superimposed with *Aa*MinCD (green: *Ec*MinD, yellow: *Ec*MinC_CTD_). The highly-conserved residues D154 in *Ec*MinD and RSGQ motif in *Ec*MinC are known to be critical for MinC-MinD interaction. (**B**) Superimposition of *Ec*MinCD with *Aa*MinCD (green: *Ec*MinD, yellow: *Ec*MinC_CTD_, blue: *Aa*MinD, orange: *Aa*MinC_CTD_). Helix3 of *Aa*MinC_CTD_ forms interface 2 with helix 8 of MinD, which is absent in *Ec*MinCD complex. (**C**) Superimposition of *Ec*MinCD with *Ec*MinDE (green: *Ec*MinD, yellow: *Ec*MinC_CTD_, magenta: *Ec*MinE). We observe that the MinE contact helix competes with MinC for binding to MinD and the interface surfaces clearly overlap^25, 26^, which provides explanation of why MinE can stimulate the release of MinC.

Previously, an *A. aeolicus* MinC_CTD_-MinD complex (*Aa*MinCD, PDB: 4V02) and an *E. coli* MinD (*Ec*MinD) dimer crystal structure had been solved (PDB: 3Q9L). Compared to *Aa*MinC_CTD_, *Ec*MinC_CTD_ and *Tm*MinC_CTD_ both lack a helix3 at its C-terminus (Fig. 1B). We superimposed our *Ec*MinC_CTD_ structure and the *Ec*MinD structure (PDB: 3Q9L) onto the *Aa*MinCD co-crystal structure (PDB: 4V02), resulting in an assembled *Ec*MinCD complex (Fig. 2A). In this *Ec*MinCD model structure, the RSGQ motif of MinC and MinD’s helix 7 make up an interaction interface as expected. In earlier studies the highly-conserved residues D154 in *Ec*MinD and RSGQ motif in *Ec*MinC were identified to be very important for MinC-MinD interaction^23, 24^ (Fig. 2A). The helix3 of *Aa*MinC_CTD_ forms interaction interface 2 with helix 8 of MinD, which is absent in both *Ec*MinC and *Tm*MinC (Fig. 2B). We superimposed the assembled *Ec*MinCD model complex (Fig. 2C) on *Ec*MinDE complex (PDB: 3R9J), which shows that the interaction interface of the *Ec*MinDE complex is more compact than that of *Ec*MinCD. More importantly, the interface surfaces clearly overlap, consistent with the previous findings^25, 26^. Thus the MinE contact helix would compete with MinC for binding to MinD, which explains why MinE could stimulate the release of MinC.

### Impact of *Aa*helix3 on cell morphology and cell growth

Next, we set to find out whether the helix 3 of *Aa*MinC is crucial for MinC’s function. We fused the *Aa*helix3 to the C-terminus of *Ec*MinC, resulting in a hybrid MinC (*Ec*MinC-*Aa*helix3) to be used in the subsequent functional experiments. SDS-PAGE and western results showed that this hybrid protein expressed well (Fig. S2) and CD analysis revealed that it was properly folded compared to the original MinC (Fig. S3). In addition, MALLS and SEC results (Fig. S4) of MBP-*Ec*MinC and MBP-*Ec*MinC-*Aa*helix3 showed that both of these two proteins formed stable dimers, which is consistent with the fact that MinC forms dimer in the *Aa*MinCD complex structure (Fig. 2B).

**Figure 3:**
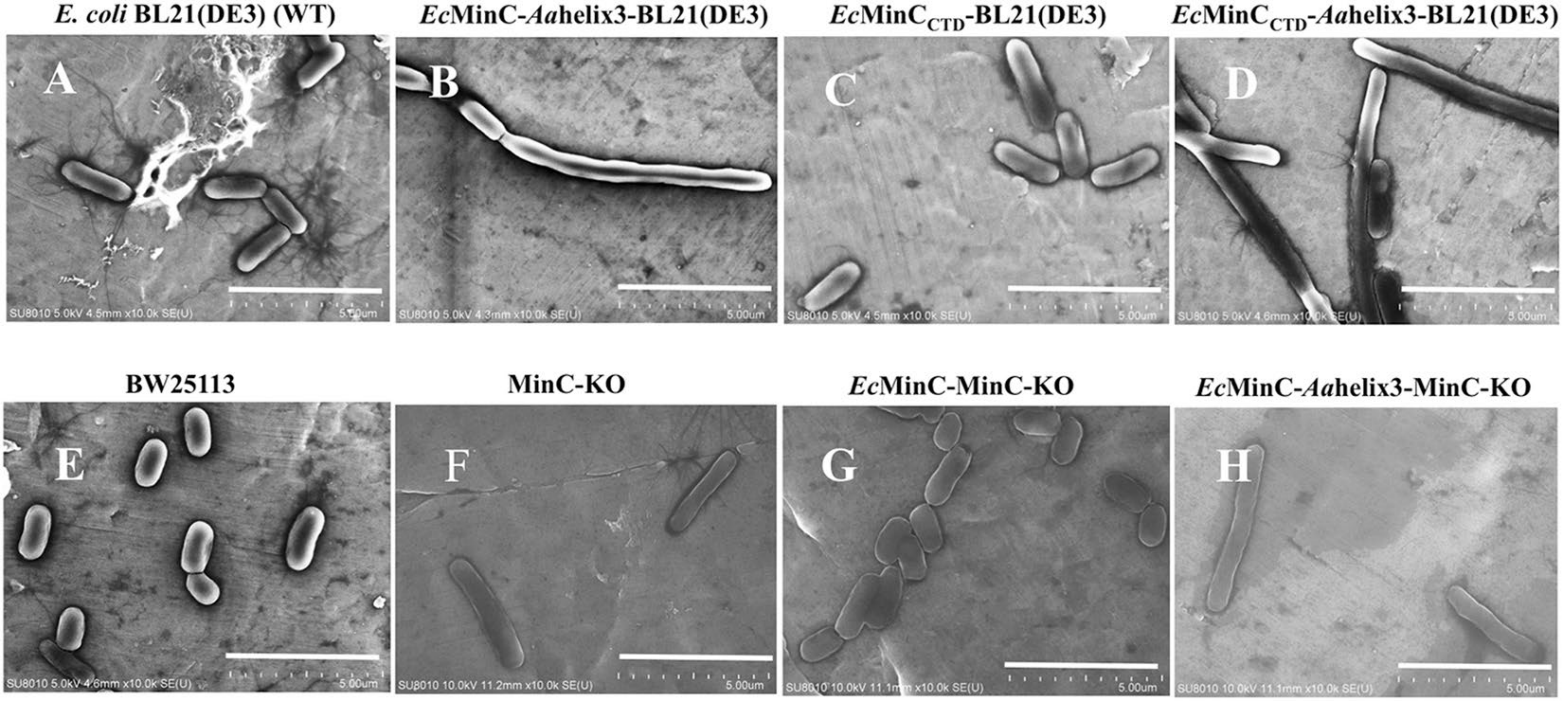
Scanning electron micrographs of derivatives of MinC expression in *E. coli* BL21(DE3) or MinC-KO. (**A**) *E. coli* BL21(DE3) (WT); (**B**) *Ec*MinC-*Aa*helix3-BL21(DE3); (**C**) *Ec*MinC_CTD_-BL21(DE3); (**D**) *Ec*MinC_CTD_-*Aa*helix3-BL21(DE3); (**E**) BW25113; (**F**) MinC-KO; (**G**) *Ec*MinC-MinC-KO; (**H**) *Ec*MinC-*Aa*helix3-MinC-KO. Cells of *Ec*MinC_CTD_-*Aa*helix3 exhibited much longer cell morphology than WT (**A**~**D**) and complementation of MinC-helix3 to MinC-KO strains could not help cells regain the ability of cell division regulation (**E**~**H**), verifying that the *Aa*helix3 influenced the normal cell division in cells. Scale bars represent 5 μm.

We anticipated that the addition of a 10-aa peptide at the C-terminal tail would have a functional consequence and alter *Ec*MinC’s characteristics in cell division regulation. To test whether the additional *Aa*helix3 affects the cell morphology of *E. coli* cells *in vivo*, we carried out SEM experiments using strains containing *Ec*MinC-*Aa*helix3 (Figs 3 and S5). As shown in the SEM images (Fig. 3A–D), the cell length of BL21(DE3) (wild-type or WT) strains was about 2.6 μm. MinC_CTD_ overexpression strains were a bit longer compared to WT strains (~3.5 μm, Fig. 3C), suggesting that without MinC_NTD_, overproduction of MinC_CTD_ could not inhibit the FtsZ filament formation at mid-cell. However, MinC_CTD_-helix3 strains exhibited much longer cell morphology than WT (>10 μm *vs* 2.6 μm), strongly suggesting that helix3 influenced the normal cell division in cells. To exclude the effect of endogenous MinC in cells, we carried out complementation experiment using MinC-KO strains and BW25113 (WT strain). As shown in Fig. 3E–H, MinC-KO cells were longer than BW25113 cells (~4 μm *vs* 1.5 μm). Consistent with our prediction, complementation of *Ec*MinC to MinC-KO strains restored normal cell length similar to BW25113 cells. Surprisingly, complementation of *Ec*MinC-*Aa*helix3 to MinC-KO strains could not help cells regain the ability of cell division regulation, in which the cells exhibited cell length of ~6 μm. Furthermore, we monitored the cell morphology of live cells of these corresponding strains through standard light micrographs and results were generally consistent to the SEM images (Fig. S6).

**Figure 4:**
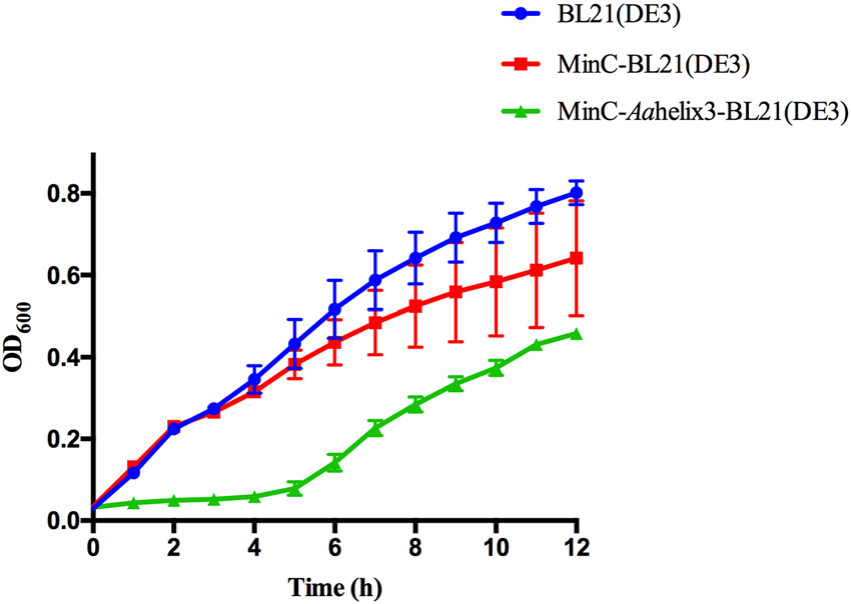
Effect of *Aa*helix3 on cell growth. Growth curves of WT *E. coli* BL21(DE3) (blue), *Ec*MinC strains (red) and *Ec*MinC-*Aa*helix3 strains (green) under aerobic conditions at 37 °C. All experiments were completed in triplicate and performed twice. The standard error of the mean was used to calculate the error bars. For those points where experimental variations are too small, their error bars are not visible.

To assess the influence of *Ec*MinC-*Aa*helix3 on the activity of *Ec*MinC, we compared growth curves of WT strain and strains overexpressing *Ec*MinC or *Ec*MinC-*Aa*helix3. Growth of the *Ec*MinC overexpression strain was slower than WT (Fig. 4), consistent with the observation that overexpression of MinC inhibits cell division^27^. Nevertheless, growth of the *Ec*MinC-*Aa*helix3 overexpression strain was slower than the *Ec*MinC overexpression strain, suggesting that helix3 increased the inhibitory activity of *Ec*MinC on cell division.

### The helix3 increases the interaction between *Ec*MinC and *Ec*MinD

**Figure 5:**
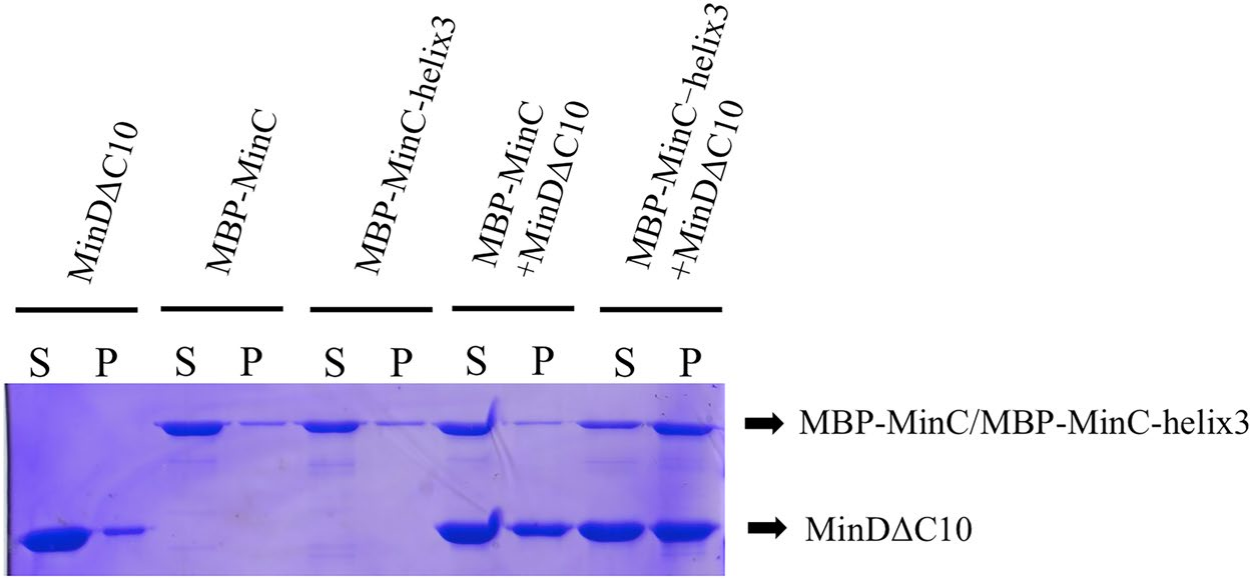
Formation of large complexes containing MinD and MinC. Mixtures containing combinations of *Ec*MinDΔC10 (12 μM) and MBP-*Ec*MinC (6 μM) or MBP-*Ec*MinC-*Aa*helix3 (6 μM), where incubated at 25 °C for 15 min and then fractionated by centrifugation. Supernatants and pellets were analyzed by SDS–PAGE and Coomassie Blue staining. This figure is a cropped gel and the full-length gel is shown in Fig. S11.

It has been reported that MinD forms copolymers with MinC in the presence of ATP^6, 28^. To further understand the protein interaction that occurs between MinC-helix3 and MinD, we studied complex formation *in vitro* using a co-pelleting assay. MinC and MinD were incubated in the absence of ATP, and then mixtures were fractionated by centrifugation. We observed that both *Ec*MinDΔC10 and MBP-*Ec*MinC, alone or together, were soluble and predominantly localized in the supernatant (Fig. 5). In addition, MBP-*Ec*MinC-*Aa*helix3 alone was also mainly found in the supernatant. However, when MBP-*Ec*MinC-*Aa*helix3 and *Ec*MinDΔC10 were incubated together, the distribution of both proteins shifted to the pellet (Fig. 5). These results demonstrate that MinC-helix3 and MinD form large oligomers *in vitro*, implying that the helix3 increases the interaction between *Ec*MinC and *Ec*MinD. Furthermore, MBP-*Ec*MinC and MBP-*Ec*MinC-*Aa*helix3 were respectively used to interfere with the interaction between MinD and MinE using a competition experiment. Our results show that MBP-*Ec*MinC is unable to replace MinE from the MinDE complex but MBP-*Ec*MinC-*Aa*helix3 successfully competes with MinE (Fig. S7) because there was almost no MinE in the elution, again suggesting that the helix3 increases the *Ec*MinC-*Ec*MinD interaction.

### Binding free energy calculation of protein complex

We next carried out binding free energy calculations to find out the relative interacting strength of *Aa*MinCD, *Ec*MinCD and *Ec*MinDE. The 6 models we used are detailed in Methods. Results (Table 1) show that without helix3, the interaction between *Aa*MinC-Δhelix3 and *Aa*MinD was much weaker compared to *Aa*MinCD (−45.86 kcal·mol^−1^ *vs* −85.54 kcal·mol^−1^). Moreover, interaction between *Ec*MinC-*Aa*helix3 and *Ec*MinD was stronger than *Ec*MinCD (−41.25 kcal·mol^−1^ *vs* −31.94 kcal·mol^−1^), indicating that the helix3 strengthened the binding of MinCD. Intriguingly, the binding of *Ec*MinC with *Ec*MinD was weaker than *Ec*MinD with *Ec*MinE (−79.96 kcal·mol^−1^ *vs* −89.21 kcal·mol^−1^), which is consistent with the fact that *Ec*MinE is able to compete with *Ec*MinC from the *Ec*MinCD complex and stimulate the release of *Ec*MinC^6^.

**Table 1:** Binding free energy calculation of protein complex.

Model Number	Illustration of model	Calculation of binding free energy	kcal·mol^−1^
Model 1	*Aa*MinCD complex	D to 2C + D^a^	−85.54
Model 2	*Aa*MinC-Δhelix3/*Aa*MinD complex	D to 2C + D	−45.86
Model 3	Assembled *Ec*MinCD complex	D to 2C + D	−31.94
Model 4	*Ec*MinC-*Aa*helix3/*Ec*MinD complex	D to 2C + D	−41.25
Model 5	*Ec*MinDE complex	2D to 2E^b^	−89.21
Model 6	Assembled *Ec*MinCD complex	2D to 2C	−79.96

D to 2C + D^a^ refers that in the model the binding free energy of one chain of MinD with the other three chains was calculated.

2D to 2E^b^ refers that in the model the binding free energy of two MinD chains with the other two MinE chains was calculated.

### Sequence analysis of MinC and MinD

**Figure 6:**
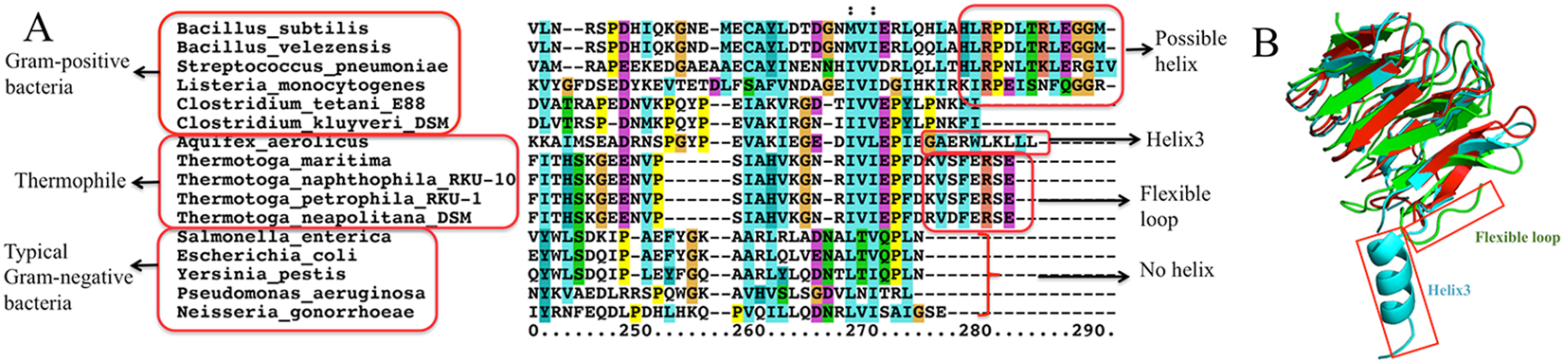
Sequence alignment of MinC from different organisms (240–291 aa) and overall structures of superimposed dimeric *Aa*MinC_CTD_, *Tm*MinC_CTD_ and *Ec*MinC_CTD_. (**A**) The C-terminal tails of MinC of the typical Gram-negative bacteria are highly similar. However, The C-terminal tails of MinC from several bacteria belonging to Thermophile (such as *A. aerolicu* and *T. maritima*) and several typical Gram-positive bacteria (such as *B. subtilis*) are longer than the Gram-negative bacteria. Alignment was performed using the program *ClustalX 2.1*. (**B**) As seen from the structures, *Tm*MinC_CTD_ has a flexible loop at its C-terminal tail and *Aa*MinC_CTD_ has a helix3 at its C-terminal tail (blue: *Aa*MinC_CTD_, green: *Tm*MinC_CTD_, red: *Ec*MinC_CTD_).

The difference in the C-terminal tail of *Aa*MinC, *Tm*MinC and *Ec*MinC led us to speculate that there may be some evolutionary relationships between MinCs from different groups of organisms. Therefore, we performed sequence analysis of MinCs (Fig. 6A, 240–291 aa) from various organisms. Results show that the C-terminal tail of MinCs of several typical Gram-negative bacteria are almost identical and lack helix3. Moreover, MinCs from Thermotogae family are conserved, which indicates that they all have a flexible loop similar to *Tm*MinC (Fig. 6A, 275–282 aa). Furthermore, there is a helix3 at *Aa*MinC’s C-terminal tail. Based on the predicted secondary structure of MinC of Gram-positive *B. subtilis* (Fig. S8), we propose that there is also a helix at *Bs*MinC’s C-terminal tail. The alignment of several MinCs from Gram-positive bacteria suggests that they all likely possess a helix at their C-terminus (Fig. 6A). Using the Neighbor-Joining method^29^, the evolutionary history is inferred and results show that the evolutionary relationships of MinC and MinD from different organisms are consistent with their sequence analysis (Figs S9, S10). To examine a possible connection amongst cell division patterns among different organisms, we have summarized the effects on cell morphology exhibited by several relevant proteins (Tables 2, Table S3). As seen in Table 2, in *E. coli* which belongs to typical Gram-negative bacteria, the cell division pattern is basically dependent on spatially regulation via *min* oscillation. *T. maritima* of Thermotogae and *A. aeolicus* of Aquificae are both members of Thermophile, whose living conditions are similar to each other at the high temperature of 95 °C^30^. However, in *A. aeolicu*, MinE is not present and its MinC has an extra helix3. Although there is also no MinE in *B. subtilis*, DivIVA plays a role in the inhibition of MinC, leading to cells undergoing a different division mechanism instead of *min* oscillation, which is a pole-anchored pattern^31^. With the help of DivIVA and MinJ, the MinCD complex remains concentrated in two polar regions, thereby preventing division from taking place near the poles. Nevertheless, neither MinC nor MinE is present in Archaea cells and the cell division pattern still remains unknown.

**Table 2:** Preliminary summary *of cell division components from different organisms*.

Organism	MinC	MinD	MinE	System Behavior
Gram-negative (*E. coli*)	Yes	Yes	Yes	Oscillation
Gram-positive (*B. subtilis*)	Yes, with possible CTD helix	Yes	No, DivIVA	Pole-anchored
Thermophile (*A. aeolicus*)	Yes, with CTD helix	Yes	No	*Likely* no oscillation without MinE
Archaea	No	Yes	No	Unknown

## Discussion

The structure of full-length *Ec*MinC has not been solved yet and only the N-terminal domain of *Ec*MinC has been determined^32^. To solve the *Ec*MinC structure, we attempted to crystallize full-length *Ec*MinC. However in the crystal structure only the CTD of *Ec*MinC was observed, consistent with the SDS-PAGE result. However, without MinC_NTD_ which possibly promotes protein folding and solubility, MinC_CTD_ construct alone was very unstable and readily precipitated (data not shown). In the *Tm*MinC structure^33^, only the C-terminal domain, but not the N-terminal domain was involved in dimer formation. In comparison, both domains of *Ec*MinC are engaged in dimerization^32^ (this work), which hints that the dimer interface of *Tm*MinC is weaker than that in *Ec*MinC.

Ghosal *et al*. determined the complex structure of *Aa*MinCD and discovered that MinC and MinD together form a new class of nucleotide-dependent, alternating copolymeric filaments^6^. A subsequent publication from the Lutkenhaus group casts reasonable doubt on the physiological relevance of these MinCD copolymers^7^. As seen from the structure of the *Aa*MinCD complex, helix3 at the C-terminal tail of *Aa*MinC and helix 8 of *Aa*MinD form interface 2, one of the two distinct interaction interfaces between the two molecules. However, this helix3 is absent in both *Ec*MinC_CTD_ and *Tm*MinC_CTD_. *A. aeolicus* is an unusual group of thermophilic bacteria, which can be found near underwater volcanoes or hot springs^30^. As seen in the secondary structure of MinC in different organisms (Fig. 6A), most typical Gram-negative bacteria do not have helix3 at the C-terminal tail of MinC. However, there is a helix3 at the C-terminus of *Aa*MinC, which belongs to the thermophilic bacteria. Furthermore, there is a coil at the C-terminal tail of the MinC in several other thermophilic bacteria including *T. maritima* (Fig. 6). The distinct structural features in different species indicate a role of helix3 (or lack of) in different cell division styles and evolutionary adaptation. The structure of *Aa*MinCD complex shows that the helix3 of *Aa*MinC forms an extra interacting interface with MinD. We speculate that in addition to its implication in cell division, this extra interaction may help stabilize the protein structure and enable *A. aerolicu* to withstand extreme environmental conditions such as high temperature.

It is widely accepted that MinC is a division inhibitor which is able to prevent Z ring formation and causes filamentation when overproduced even in the absence of MinD and MinE^34^. The C-terminal domain of MinC interacts with MinD, while its N-terminal domain directly inhibits FtsZ filamentation^35^. Our SEM experiments show that *Ec*MinC_CTD_-helix3 strains exhibited longer cells than *Ec*MinC_CTD_ strains (Fig. 3). Moreover, our cell growth curves demonstrate that the *Aa*helix3 increased MinC’s inhibitory activity *in vivo* (Fig. 4). Based on the literature and our findings, we propose that the fusion of helix3 to *Ec*MinC leads to disruption of cell division because there exists stronger interaction in MinC-helix3/MinD complex so that MinE cannot trigger the release of MinC. Consistently, the co-pelleting results show that MinC-helix3 could form large complexes with MinD *in vitro* (Fig. 5). SEM images (Fig. 3) reveal that complementation of *Ec*MinC could rescue the loss of cell division inhibition in MinC-KO strain, while complementation of *Ec*MinC-*Aa*helix3 could not rescue the inhibitory ability. When MinC was transformed into MinC-KO, the induced MinC helped cells to undergo normal cell division. However, when *Ec*MinC-*Aa*helix3 was transformed into MinC-KO, the helix3 of MinC interfered MinE’s function of triggering the release of MinC, hence impairing cells’ normal cell division.

In *E. coli*, MinC and MinD undergo a rapid pole-to-pole oscillation in response to MinE, which causes a periodic block of the polar division sites^36, 37^. MinE is reported to bind to MinCD to trigger the release of MinC and stimulates the ATPase activity of MinD^4^. Afterwards, MinD is dissociated from the membrane, thus the central division site is protected from inhibition by MinCD. The superposition of *Ec*MinDE and *Ec*MinCD structures reveals that the MinE contact helix would compete with MinC for binding to MinD since the interface surfaces clearly overlap (Fig. 2C)^6^, which is consistent with previous findings^25, 26^. In addition, our binding free energy calculation of *Ec*MinCD and *Ec*MinDE (Table 1) reveals that there is a stronger interaction of *Ec*MinDE than *Ec*MinCD complex, leading to our speculation that the interaction of *Ec*MinCD complex is weaker than *Aa*MinCD due to solely one interaction interface, which is consistent with our negative results of pull-down experiments using *Ec*MinC and *Ec*MinD *in vitro* (data not shown). Only in this case is *Ec*MinE able to replace MinC from the MinCD complex and stimulate MinC’s dissociation from the membrane. As shown in Table 2, in Gram-negative bacteria such as *E. coli*, there is no helix3 at the C-terminal tail of *Ec*MinC and *Ec*MinE is present. However, in Gram-positive bacteria, DivIVA, instead of MinE, plays a role in inhibition of MinCD^38^ and cell undergoes a different division mechanism. There is no MinE and DivIVA in *A. aerolicu*, we speculate that there also exists no *min* oscillation in *A. aerolicu* cell cycle due to the intimate interaction between *Aa*MinCD. Moreover, in Archaea we find that both MinC and MinE are lost and cells would undergo a totally different, although yet unknown, division mechanism.

In summary, in this study we determined the crystal structure of the dimeric C-terminal domain of *Ec*MinC. Through the structural alignment of *Aa*MinC_CTD_, our *Ec*MinC_CTD_ and *Tm*MinC_CTD_, we were intrigued by the fact that there exists an extra helix3 at *Aa*MinC_CTD_’s C-terminal tail, which forms a tight interaction with MinD. SEM experiments and cell growth assays demonstrate that *Aa*helix3 fused at the C-terminus of *Ec*MinC interfered normal cell division. Combined with results of binding free energy calculation and co-pelleting assay, we conclude that the interaction of *Aa*MinCD complex is stronger than *Ec*MinCD. Sequence analysis of MinC and MinD in different organisms and comparison of cell division pattern with *B. subtilis* have enabled us to propose that *T. maritima*, *A. aerolicu* and Archaea undergo different cell division cycles. The interaction between MinE and helix3 of MinC (or lack thereof) in different organisms provides a clue for explaining the different mechanisms in cell divisions among Bacteria and Archaea, although the detailed evolutionary changes in cell division pattern and mechanism remain to be investigated.

## Methods

### Strain materials and plasmid construction

Standard methods were used for plasmid construction. The plasmids utilized in this study are listed in Table S1. The coding sequence for MinC was amplified from *E. coli* K12 and inserted into pET22b expression plasmid through *Nde*Ι and *Xho*Ι to express a soluble construct containing a 6 × His at the C-terminus. For SEM and cell growth experiments, in order to obtain fusion protein of *Ec*MinC-*Aa*helix3 and *Ec*MinC_CTD_-*Aa*helix3, the sequence of helix3 from *Aa*MinC_CTD_ was fused to the C-terminus of *Ec*MinC/*Ec*MinC_CTD_ using two-step site-directed mutagenesis. The resulting sequence was inserted into pET22b through *Nde*Ι and *Xho*Ι. For constructs used in MinCD co-pelleting assay and competition experiment, genes encoding MBP-*Ec*MinC or MBP-*Ec*MinC-*Aa*helix3 were inserted into a modified pET-28b vector (mod-pET28b). The N-terminal His-tag in the original pET-28b vector was replaced by an MBP-tag through *Nco*Ι and *Nde*Ι and *Ec*MinC/*Ec*MinC-*Aa*helix3 were inserted through *Nde*Ι and *Xho*Ι. After insertion, *Ec*MinC or *Ec*MinC-*Aa*helix3 was expressed with MBP at its N-terminus and with or without His-tag at its C-terminus. *Ec*MinD was truncated by 10 amino acids at the C-terminus (ΔC10) to increase solubility by removing the amphipathic helix (MTS) and inserted into pET22b through *Nde*Ι and *Xho*Ι to express MinDΔC10-6 × His protein. Gene encoding *Ec*MinE was inserted into pET22b through *Nde*Ι and *Xho*Ι and a strep-tag was fused to the C-terminus of MinE through site-directed mutagenesis. Bacterial strain TOP10 was used for general cloning and plasmid maintenance. BL21(DE3) strain was used for protein expression, SEM and cell growth experiments. Strains of *E. coli* BW25113 (wild-type) and MinC-KO were obtained from *E. coli* Genetic Resources at Yale CGSC (http://cgsc.biology.yale.edu/KeioList.php) and used for SEM experiments.

### Protein expression and purification

For protein preparation and crystallization trials, BL21(DE3) *E. coli* cells were transformed by plasmid of pET22b-MinC and cultivated in LB medium. Cells were induced at OD_600 _~ 0.8 by 0.5 mM isopropy-β-D-thiogalactoside (IPTG) and cultured at 16 °C for 20 h and harvested for disruption by sonication in buffer containing 20 mM Tris-HCl, pH 7.5, 200 mM NaCl (buffer A). After centrifugation, the supernatant was mixed with 2 ml of Ni-agarose resin. Next, beads were washed with 50 ml buffer A containing 30 mM imidazole and 300 mM imidazole was used to elute protein. Eluted samples were pooled and subjected to a size-exclusion chromatography (HiLoad superdex16/60 S200, GE Healthcare) using an AKTA avant system (GE Healthcare) equilibrated with buffer containing 20 mM Tris-HCl, pH 8.0, 100 mM NaCl. Fractions containing the desired protein were pooled and concentrated for crystallization screening. For proteins used in co-pelleting assays, MBP-*Ec*MinC, MBP-*Ec*MinC-*Aa*helix3 and MinDΔC10 were expressed and purified in a similar way except that proteins were purified in buffer containing 20 mM Tris-HCl, pH 7.5, 150 mM NaCl (buffer B). The purified proteins were concentrated and stored with 20% glycerol at −80 °C until use. Proteins were analyzed by SDS-PAGE and visualized by Coomassie Brilliant Blue (CBB) staining. For western blotting analysis of the *Ec*MinC-*Aa*helix3 construct, eluted protein from Ni-agarose resin were subjected to SDS-polyacrylamide gel electrophoresis, transferred onto PVDF membrane and blocked for 1 h with TBST containing 5% milk. A mouse monoclonal anti-His-HRP conjugated antibody (Tianjin Sungene Biotech) was used for primary antibody and goat alkaline phosphatase-conjugated anti-mouse secondary antibody was used for protein detection.

### Crystallization and X-Ray Diffraction

Soluble MinC protein (15 mg/ml) was crystallized by hanging-drop vapor diffusion method at 20 °C mixing 2 μL protein solution with 2 μL reservoir solution containing 0.1 M Tris-HCl, pH 7.2, 0.44 M sodium/potassium L-(+)-tartrate. Over time, MinC protein was degraded into two separate domains (MinC_NTD_ and MinC_CTD_) and needle-shaped crystals of MinC_CTD_ were grown over a period of 3–6 weeks. Crystals were cryoprotected in reservoir solution with 20–30% glycerol and flash frozen in liquid nitrogen. X-Ray diffraction data were collected at the beam line 13B1 of National Synchrotron Radiation Research Center (Hsinchu, Taiwan) and processed with HKL-3000^39^.

### Structure determination, refinement and analysis

The structure was determined by molecular replacement using *Phaser* ^40^ using the C-terminal domain structure of *Tm*MinC (PDB: 1HF2) as a search model. Model building and refinement were performed using *Coot* ^41^ and *phenix.refine* ^42^, respectively. The figures were generated using *PyMOL* (http://www.pymol.org/). The atomic coordinates and structure factors have been deposited in the Protein Data Bank as entry 5XDM. The data collection and refinement statistics are summarized in Table S2.

### Scanning electron microscopy (SEM)

For scanning electron microscopy sample preparation, overnight cultures of strains containing MinC derivatives were diluted to OD_600_ 0.05 in fresh LB at 37 °C. Strains were induced after 4 h with 1 mM IPTG and cultured overnight at 16 °C under 150 rpm shaking. Cells were then harvested by centrifugation for 10 min at 5000 rpm. After wash three times using PBS (pH 7.4), cells were then dehydrated through 70% acetone solution and dropped onto foil which was glued onto a metal specimen holder after the cells had dried. The SEM images of cells were obtained using a SU8010 Scanning Electron Microscope (Hitachi). The cell length was measured (over 50 per each sample) for statistical analysis using Graphpad Prism 6 (Graphpad software, Inc).

### Cell growth conditions

Different MinC strains were cultured in LB overnight and diluted to the same OD_600_. 1 mM IPTG was added to cultures of each strain. The cultures were then incubated on 96-well plates with shaking at 37 °C for 12 h and cell growth was monitored by taking OD_600_ measurements hourly. During measurements, the optical path length is approximately 5 mm. Each sample was assayed in triplicate.

### Co-pelleting assay

For the MinCD co-pelleting assay, MBP-*Ec*MinC, MBP-*Ec*MinC-*Aa*helix3 and MinDΔC10 proteins were used. Proteins were purified as described before. Mixtures (50 μL) in assembly buffer (50 mM MES, pH 6.5, 100 mM KCl, 10 mM MgCl_2_) containing MinD (12 μM) and MinC (6 μM) were incubated for 15 min at 25 °C, then centrifuged at 14 000 × g for 30 min. After that, the supernatant and pellets were analyzed by SDS-PAGE. Control experiments were carried out in a similar way using individual protein.

### Binding free energy calculation

After 5 ns MD simulation, stable models of protein complex were obtained. The molecular mechanics Poisson-Boltzmann surface area (MM-PBSA) method^43–45^ implemented in the AMBER14 package was employed to calculate the binding free energy of protein complex. The binding free energy was obtained through calculating the free energy differences of ligand, receptor, and their complex as follows:

$${\rm{\Delta }}{G}_{binding}={G}_{complex}-{G}_{ligand}-{G}_{receptor}$$

The 6 models (Table 1) we used were described as bellows: model 1: *Aa*MinCD complex (PDB: 4V02); model 2: the helix3 of *Aa*MinC in *Aa*MinCD complex was eliminated to constitute *Aa*MinC-Δhelix3/*Aa*MinD complex; model 3: assembled *Ec*MinCD complex using *Ec*MinC_CTD_ (this work) and *Ec*MinD (PDB: 3Q9L) superimposed with *Aa*MinCD; model 4: the *Aa*helix3 was added to the C-terminus of *Ec*MinC to constitute *Ec*MinC-*Aa*helix3/*Ec*MinD complex. In models 1–4, the binding free energy of one chain of MinD with the other three chains in the complex was calculated. In model 5, *Ec*MinDE complex (PDB: 3R9J), the binding free energy of two MinD chains with two MinE chains was calculated. In model 6, assembled *Ec*MinCD complex (same as model 3), the binding free energy of two MinD chains with two MinC chains was calculated.

## Electronic supplementary material

Supplementary Information

## References

[1] Young KD (2006). The selective value of bacterial shape. Microbiol. Mol. Biol. Rev..

[2] Deboer PAJ, Crossley RE, Rothfield LI (1989). A division inhibitor and a topological specificity factor coded for by the minicell locus determine proper placement of the division septum in *E. coli*. Cell.

[3] Varma A, Huang KC, Young KD (2008). The Min system as a general cell geometry detection mechanism: Branch lengths in Y-shaped *Escherichia coli* cells affect Min oscillation patterns and division dynamics. J. Bacteriol..

[4] Park KT (2011). The Min Oscillator Uses MinD-Dependent Conformational Changes in MinE to Spatially Regulate Cytokinesis. Cell.

[5] Hu ZL, Lutkenhaus J (2000). Analysis of MinC reveals two independent domains involved in interaction with MinD and FtsZ. J. Bacteriol..

[6] Ghosal D, Trambaiolo D, Amos LA, Lowe J (2014). MinCD cell division proteins form alternating copolymeric cytomotive filaments. Nat. Commun..

[7] Park KT, Du S, Lutkenhaus J (2015). MinC/MinD copolymers are not required for Min function. Mol. Microbiol..

[8] Hu Z, Lutkenhaus J (1999). Topological regulation of cell division in *Escherichia coli* involves rapid pole to pole oscillation of the division inhibitor MinC under the control of MinD and MinE. Mol. Microbiol..

[9] Raskin DM, de Boer PA (1999). Rapid pole-to-pole oscillation of a protein required for directing division to the middle of *Escherichia coli*. Proc. Natl. Acad. Sci. USA.

[10] Marston AL, Errington J (1999). Selection of the midcell division site in Bacillus subtilis through MinD-dependent polar localization and activation of MinC. Mol. Microbiol..

[11] Marston AL, Thomaides HB, Edwards DH, Sharpe ME, Errington J (1998). Polar localization of the MinD protein of Bacillus subtilis and its role in selection of the mid-cell division site. Genes Dev..

[12] Bramkamp M (2008). A novel component of the division-site selection system of Bacillus subtilis and a new mode of action for the division inhibitor MinCD. Mol. Microbiol..

[13] Patrick JE, Kearns DB (2008). MinJ (YvjD) is a topological determinant of cell division in Bacillus subtilis. Mol. Microbiol..

[14] Woese CR, Kandler O, Wheelis ML (1990). Towards a natural system of organisms: proposal for the domains Archaea, Bacteria, and Eucarya. Proc. Natl. Acad. Sci. USA.

[15] Bang C, Schmitz RA (2015). Archaea associated with human surfaces: not to be underestimated. Fems Microbiol. Rev..

[16] Makarova KS, Yutin N, Bell SD, Koonin EV (2010). Evolution of diverse cell division and vesicle formation systems in Archaea. Nat. Rev. Microbiol..

[17] Bernander R (2003). The archaeal cell cycle: current issues. Mol. Microbiol..

[18] Bernander R, Lundgren M, Ettema TJG (2010). Comparative and functional analysis of the archaeal cell cycle. Cell Cycle.

[19] Guiral M (2012). The hyperthermophilic bacterium Aquifex aeolicus: from respiratory pathways to extremely resistant enzymes and biotechnological applications. Adv. Microb. Physiol..

[20] Blamey JM, Adams MW (1994). Characterization of an ancestral type of pyruvate ferredoxin oxidoreductase from the hyperthermophilic bacterium, Thermotoga maritima. Biochemistry.

[21] Schafer G (2004). Extremophilic archaea and bacteria - Introduction. J. Bioenerg. Biomembr..

[22] Cordell SC, Anderson RE, Lowe J (2001). Crystal structure of the bacterial cell division inhibitor MinC. EMBO J..

[23] Zhou H, Lutkenhaus J (2005). MinC mutants deficient in MinD- and DicB-mediated cell division inhibition due to loss of interaction with MinD, DicB, or a septal component. J. Bacteriol..

[24] Wu W, Park KT, Holyoak T, Lutkenhaus J (2011). Determination of the structure of the MinD-ATP complex reveals the orientation of MinD on the membrane and the relative location of the binding sites for MinE and MinC. Mol. Microbiol..

[25] Hu Z, Saez C, Lutkenhaus J (2003). Recruitment of MinC, an inhibitor of Z-ring formation, to the membrane in *Escherichia coli*: role of MinD and MinE. J. Bacteriol..

[26] Lackner LL, Raskin DM, de Boer PA (2003). ATP-dependent interactions between *Escherichia coli* Min proteins and the phospholipid membrane *in vitro*. J. Bacteriol..

[27] Shiomi D, Margolin W (2007). The C-terminal domain of MinC inhibits assembly of the Z ring in *Escherichia coli*. J. Bacteriol..

[28] Conti J, Viola MG, Camberg JL (2015). The bacterial cell division regulators MinD and MinC form polymers in the presence of nucleotide. FEBS Lett..

[29] Saitou N, Nei M (1987). The neighbor-joining method: a new method for reconstructing phylogenetic trees. Mol. Biol. Evol..

[30] Deckert G (1998). The complete genome of the hyperthermophilic bacterium Aquifex aeolicus. Nature.

[31] Harry EJ, Lewis PJ (2003). Early targeting of Min proteins to the cell poles in germinated spores of Bacillus subtilis: evidence for division apparatus-independent recruitment of Min proteins to the division site. Mol. Microbiol..

[32] An JY (2013). Crystal structure of the N-terminal domain of MinC dimerized via domain swapping. J. Synchrotron. Radiat..

[33] Cordell SC, Anderson RE, Lowe J (2001). Crystal structure of the bacterial cell division inhibitor MinC. EMBO J..

[34] Hu ZL, Lutkenhaus J (1999). Topological regulation of cell division in *Escherichia coli* involves rapid pole to pole oscillation of the division inhibitor MinC under the control of MinD and MinE. Mol. Microbiol..

[35] Johnson JE, Lackner LL, de Boer PAJ (2002). Targeting of (D)MinC/MinD and (D)MinC/DicB complexes to septal rings in *Escherichia coli* suggests a multistep mechanism for MinC-mediated destruction of nascent FtsZ rings. J. Bacteriol..

[36] Corbin BD, Yu XC, Margolin W (2002). Exploring intracellular space: function of the Min system in round-shaped *Escherichia coli*. EMBO J..

[37] Lutkenhaus J (2008). Min oscillation in bacteria. Adv. Exp. Med. Biol..

[38] Eswaramoorthy P (2011). Cellular Architecture Mediates DivIVA Ultrastructure and Regulates Min Activity in Bacillus subtilis. mBio.

[39] Otwinowski Z, Minor W (1997). Processing of X-ray diffraction data collected in oscillation mode. Methods Enzymol..

[40] McCoy AJ (2007). Phaser crystallographic software. J. Appl. Crystallogr..

[41] Emsley P, Cowtan K (2004). Coot: model-building tools for molecular graphics. Acta Crystallogr. Sect. D-Biol. Crystallogr..

[42] Afonine PV (2012). Towards automated crystallographic structure refinement with phenix.refine. Acta Crystallogr. Sect. D-Biol. Crystallogr..

[43] Kollman PA (2000). Calculating structures and free energies of complex molecules: combining molecular mechanics and continuum models. Acc. Chem. Res..

[44] Kuhn B, Kollman PA (2000). Binding of a diverse set of ligands to avidin and streptavidin: an accurate quantitative prediction of their relative affinities by a combination of molecular mechanics and continuum solvent models. J. Med. Chem..

[45] Kuhn B, Gerber P, Schulz-Gasch T, Stahl M (2005). Validation and use of the MM-PBSA approach for drug discovery. J. Med. Chem..

