# Supplementary material for: Characterization of C-terminal structure of MinC and its implication in evolution of bacterial cell division
Source: Sci Rep. 2017 Aug 8;7:7627. doi: 10.1038/s41598-017-08213-5 (PMC5548801; doi:10.1038/s41598-017-08213-5)
Supplement: Supplementary file 1 — Supplementary Information [file 41598_2017_8213_MOESM1_ESM.pdf]

Supplementary Information

Characterization of C-terminal structure of MinC and its implication in evolution of bacterial cell division

Shaoyuan Yang[1], Qingya Shen[1], Shu Wang[1], Chen Song[1], Zhen Lei[1], Shengnan Han[1], Xiaoying Zhang[1], Jimin Zheng[1]*, Zongchao Jia[2]*

[1]College of Chemistry, Beijing Normal University, Beijing, 100875, China. [2]Department of Biomedical and Molecular Sciences, Queen's University, Kingston, Ontario, K7L 3N6, Canada. Correspondence and requests for materials should be addressed to J.Z. (email: jimin_z@bnu.edu.cn) or Z.J. (email: jia@queensu.ca)

Shaoyuan Yang and Qingya Shen contributed equally to this work and should be considered as co-first authors.

Supplementary Methods

CD analysis

*Ec*MinC and *Ec*MinC-*Aa*helix3 samples at a concentration of 270 μM were prepared in ddH_2O. Far-UV CD spectra were recorded on a Jasco spectropolarimeter with a 1 mm path length at 20 ℃. Spectra were an average of 5 scans over the wavelength from 200 nm to 260 nm. The secondary structure content of each sample was obtained using the CDNN prediction program[1].

Multi-angle laser light scattering (MALLS)

The molecular weight range and polydispersity of MBP-*Ec*MinC and MBP-*Ec*MinC-*Aa*helix3 were analyzed by SEC equipped with a MALLS detector. Protein samples were prepared at a concentration of 2 mg/ml and filtered through a 0.22-μm filter. 200 μl of the protein sample was loaded onto KW-G and KW-803 columns (Shimadzu) at a flow rate of 0.5 ml/min. The protein signals were detected by a DAWN HELEOS-II light scattering detector and an Optilab rex refractive index detector (Wyatt Technology). The molecular weight was calculated by peaks integration (ASTRA software version 5.3.4.13). Each sample was carried out independently and repeated at least three times.

Live cell microscopy

Plasmids contained MinC or its derivatives were transformed into BL21(DE3). Overnight cultures of strains were diluted to OD_{600} 0.05 in fresh LB containing ampicillin or kanamycin at 37 ℃. Strains were induced after 4 h with 1 mM IPTG and cultured overnight at 16 ℃ under 150 rpm shaking. Cellular phenotypes were examined by phase-contrast microscopy using an oil immersion objective (100×).

Competition experiment of MBP-MinC/MBP-MinC-*Aa*helix3 with MinE

For competition experiment, MinDΔC10-His, MBP-*Ec*MinC, MBP-*Ec*MinC-*Aa*helix3 and MinE-strep proteins were used. MinDΔC10-His protein was purified as described in Methods. MinE-strep was purified using *Strep* Sepharose resins with standard protocol and eluted with buffer containing 2.5 mM D-Desthiobiotin. MBP-*Ec*MinC and MBP-*Ec*MinC-*Aa*helix3 proteins without His-tag were purified using an amylose column and eluted with buffer containing 10 mM maltose. Mixture containing MinDΔC10-His and MinE-Strep were incubated for 2 h and loaded onto a Ni-agarose column. After washing with 50 column volume of buffer B (20 mM Tris, pH 7.5, 150 mM NaCl), MBP-*Ec*MinC or MBP-*Ec*MinC-*Aa*helix3 protein (at equal amount) was loaded onto the Ni-agarose column to replace MinE from MinDE complex. After washing for another 50 column volume of buffer B, protein was eluted with buffer B containing 300 mM Imidazole. The amount of MinE in the eluted buffer was determined by using a mouse monoclonal anti-strep-tag antibody. Control experiments were carried out using the same procedures without MBP-*Ec*MinC or MBP-*Ec*MinC-*Aa*helix3. Another control experiment was carried out in order to confirm that MinE-strep had no non-specific binding to Ni-agarose column.

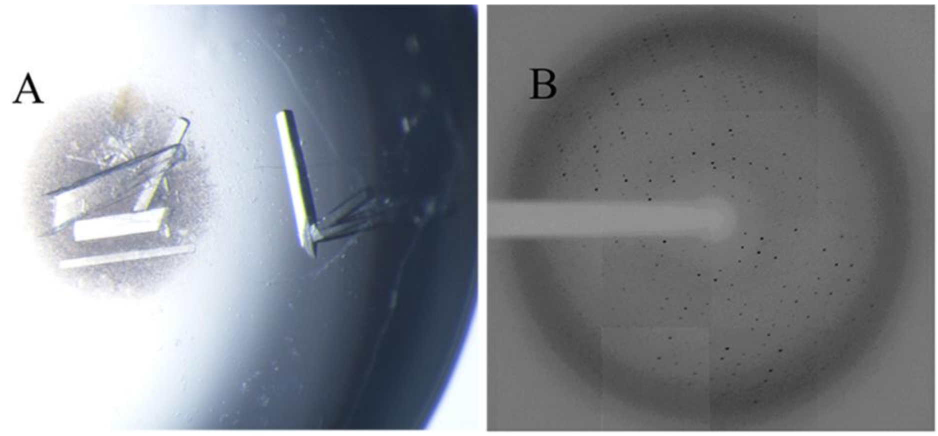

Figure S1. Crystals of *Ec*MinC$_{CTD}$ and its typical diffraction image. (A) Needle-shaped crystals of MinC$_{CTD}$ grew to the maximum size of 0.2~0.3 mm after 3~6 weeks. Afterwards, crystals were harvested and cryoprotected in reservoir solution with 20%-30% glycerol and flash frozen in liquid nitrogen. (B) The typical X-ray diffraction image of MinC$_{CTD}$ crystals. Data were processed and structure was determined at 3.0 Å resolution.

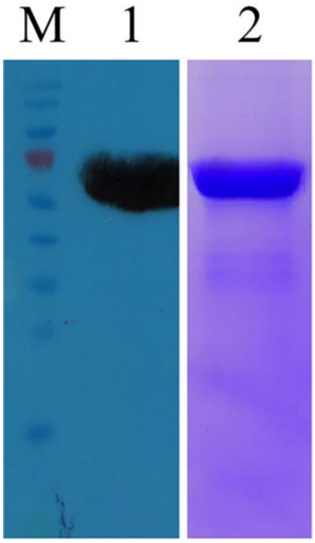

Figure S2. SDS-PAGE analysis of *Ec*MinC-*Aa*helix3 protein purified by Ni-NTA column from bacterial lysate. MW: molecular weight marker; Lane 1: western blotting analysis of the duplicated gel transferred onto PVDF membrane and probed with a mouse monoclonal anti-His-HRP conjugated antibody. Lane 2: SDS-PAGE of the eluted protein fraction from His-Trap HP column. Sample loaded onto Lane 2 was 5 times of that on Lane 1.

A

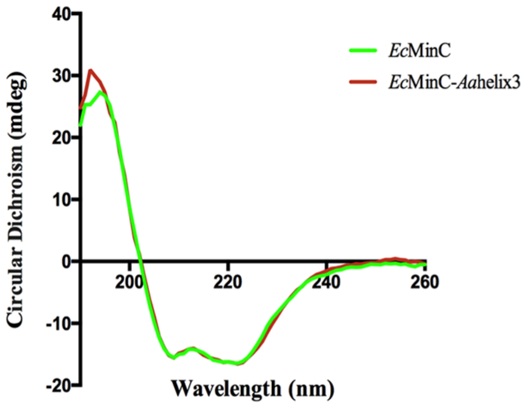

B

	Alpha (%)	Beta (%)	Random coil (%)
*Ec*MinC	22.6	43.9	41.6
*Ec*MinC-*Aa*helix3	23.5	43.6	41

Figure S3. Circular Dichroism spectra of *Ec*MinC and *Ec*MinC-*Aa*helix3. (**A**) Circular Dichroism spectra of *Ec*MinC (green) and *Ec*MinC-*Aa*helix3 (red), indicating well-folded structures. (**B**) The percentage of secondary structure content was estimated from the CD spectra using the CDNN prediction program. Results show that the percentage of alpha helix in *Ec*MinC-*Aa*helix3 was a bit more than that in *Ec*MinC.

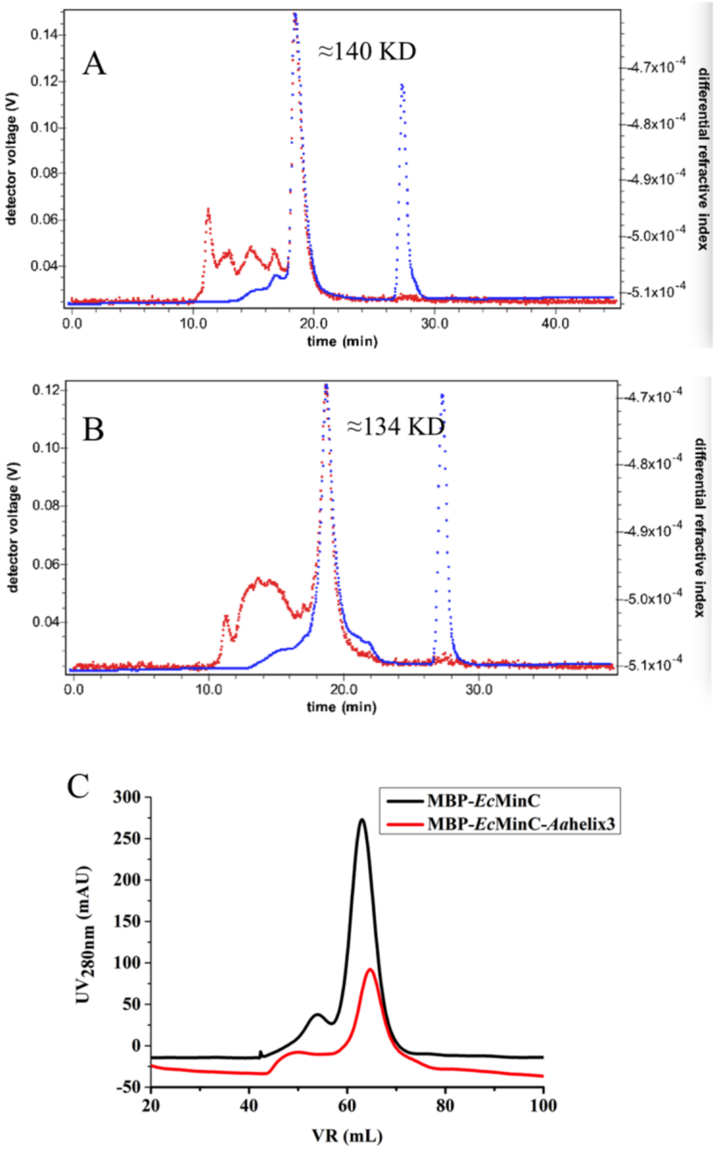

Figure S4. MALLS and SEC results of MBP-*Ec*MinC and MBP-*Ec*MinC-*Aa*helix3. (**A~B**) MALLS results of MBP-*Ec*MinC (A, ~140 KD) and MBP-*Ec*MinC-*Aa*helix3 (B, ~134 KD). (**C**) SEC profiles of MBP-*Ec*MinC (black) and MBP-*Ec*MinC-*Aa*helix3 (red). A major peak with an apparent molecular weight of 134 kD corresponding to dimer MBP-*Ec*MinC/MBP-*Ec*MinC-*Aa*helix3 was observed. Results show that both of MBP-*Ec*MinC and MBP-*Ec*MinC-*Aa*helix3 proteins formed stable dimers.

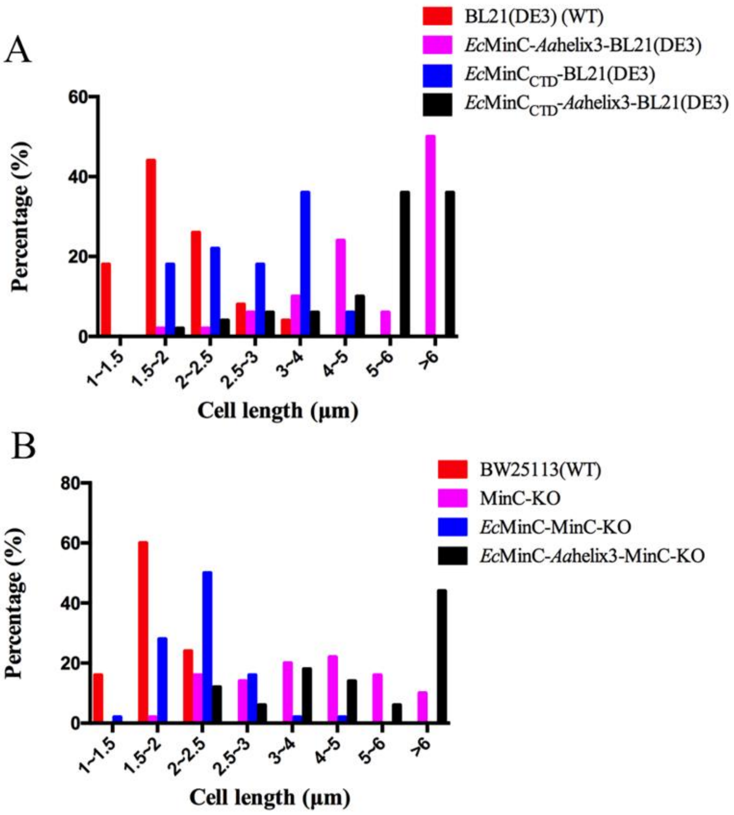

Figure S5. Statistical analysis of SEM data in Figure 3. (**A**) BL21(DE3) (WT, red); *Ec*MinC-*Aa*helix3-BL21(DE3) (magenta); *Ec*MinC$_{CTD}$-BL21(DE3) (blue); *Ec*MinC$_{CTD}$-*Aa*helix3-BL21(DE3) (black). (**B**) BW25113 (WT, red); MinC-KO (magenta); *Ec*MinC-MinC-KO (blue); *Ec*MinC$_{CTD}$-*Aa*helix3-MinC-KO (black).

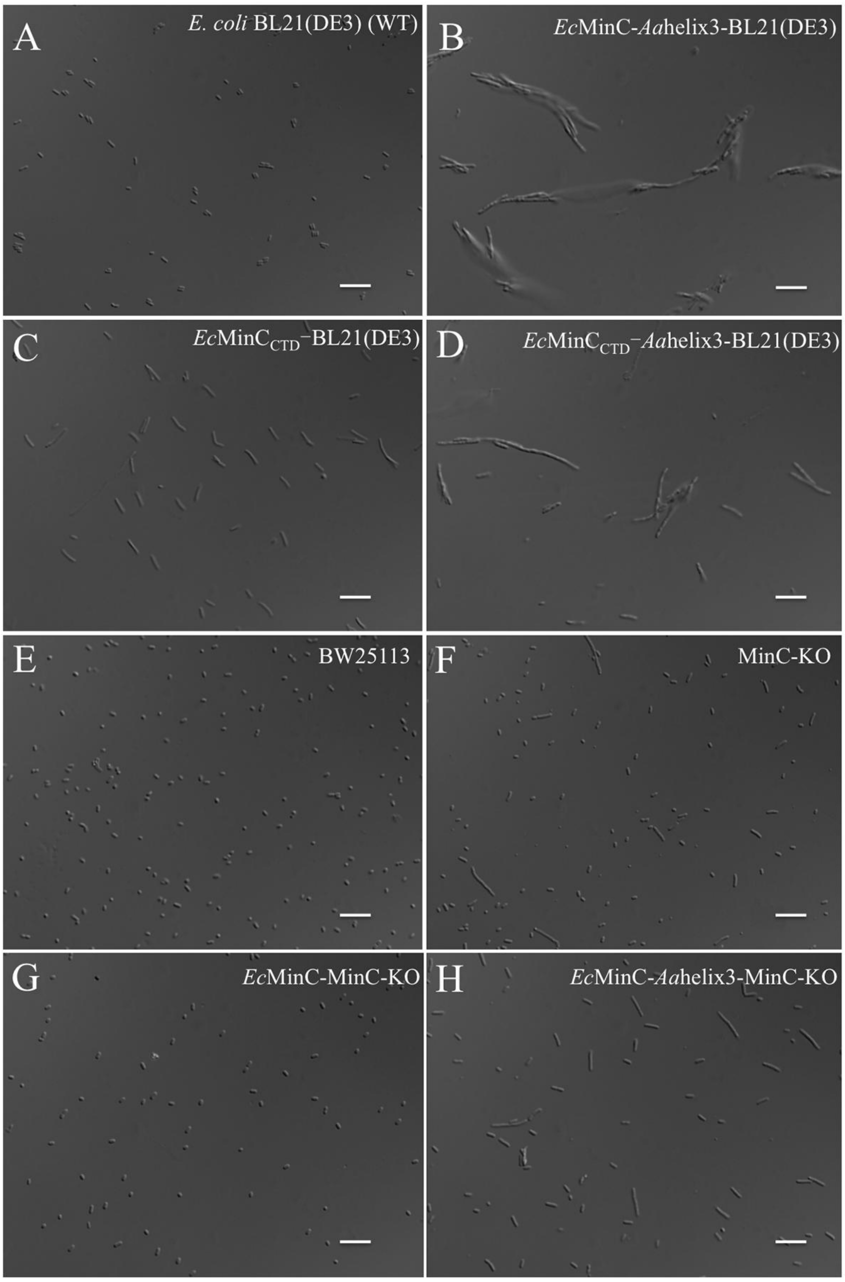

Figure S6. Standard light micrographs of MinC derivatives expressed in *E. coli* BL21(DE3) or MinC-KO. (**A**) *E. coli* BL21(DE3) (WT); (**B**) *Ec*MinC-*Aa*helix3-BL21(DE3); (**C**) *Ec*MinC$_{CTD}$-BL21(DE3); (**D**) *Ec*MinC$_{CTD}$-*Aa*helix3-BL21(DE3); (**E**) BW25113; (**F**) MinC-KO; (**G**) *Ec*MinC-MinC-KO; (**H**) *Ec*MinC-*Aa*helix3-MinC-KO. Consistent with the SEM results, cells of *Ec*MinC$_{CTD}$-*Aa*helix3 exhibited much longer cell morphology than WT (**A**~**D**) and complementation of MinC-helix3 to MinC-KO strains could not help cells regain the ability of cell division regulation (**E**~**H**), verifying that the *Aa*helix3 influenced the normal cell division in cells. Scale bars represent 10 μm.

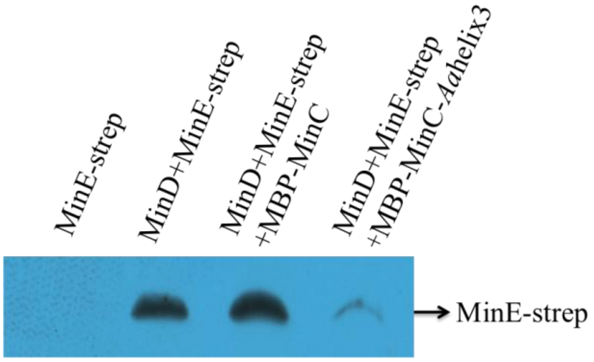

Figure S7. Competition experiment of MBP-MinC/MBP-MinC-*Aa*helix3 with MinE. The control experiment was done in order to confirm that MinE-strep has no non-specific binding to the Ni-agarose column. Results show that MBP-*Ec*MinC could not replace MinE from MinDE complex but MBP-*Ec*MinC-*Aa*helix3 could because there was almost no MinE in the elution, suggesting that the helix3 increases the interaction between *Ec*MinC and *Ec*MinD.

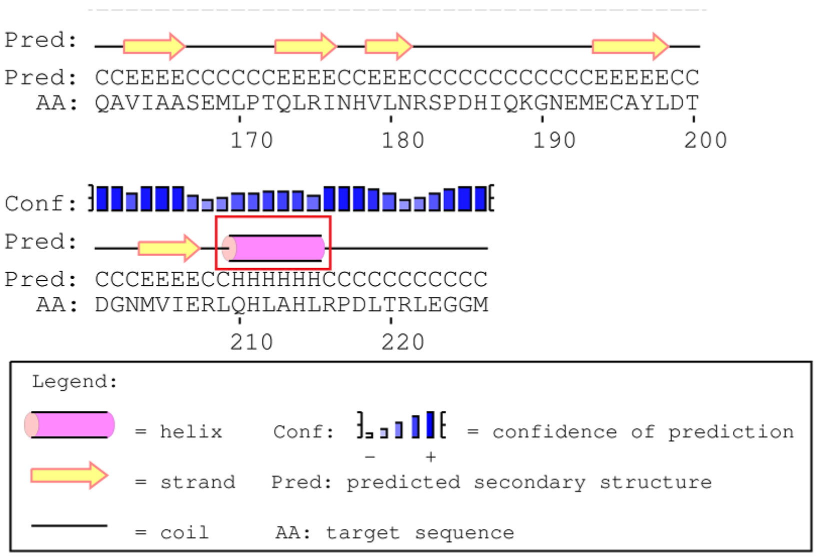

Figure S8. Predicted secondary structure of the C-terminal fragment of *B. subtilis* MinC (160 aa-226 aa). The secondary structure of *Bs*MinC (160-226 aa) was predicted using a server from Bioinformatic Group of UCL Department of Computer Science. According to its predicted secondary structure, there is an α-helix from 210 aa to 215 aa at *Bs*MinC's C-terminal tail.

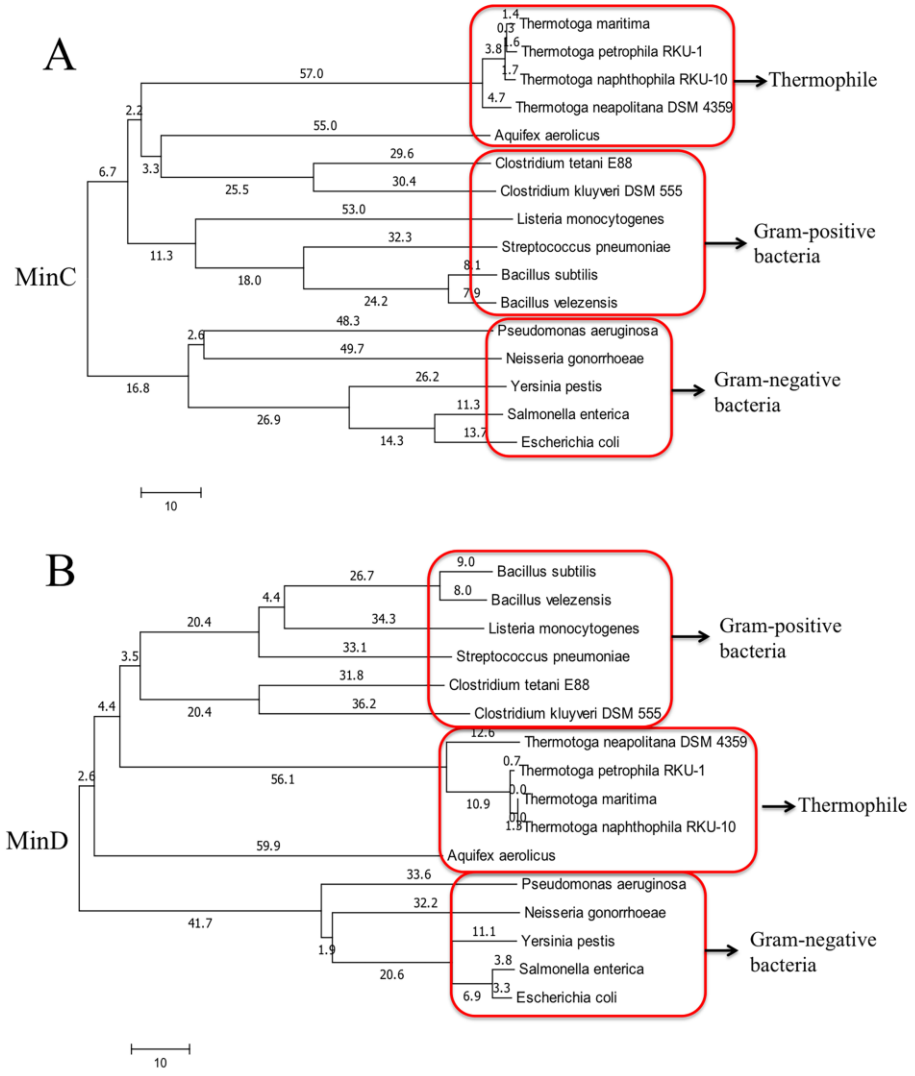

Figure S9. Evolutionary relationships of MinC and MinD. The evolutionary history was inferred using the Neighbor-Joining method. The trees are drawn to scale, with branch lengths (next to the branches) in the same units as those of the evolutionary distances used to infer the phylogenetic tree. The evolutionary distances were computed using the number of differences method and are in the units of the number of amino acid differences per sequence. All positions containing gaps and missing data were eliminated. Evolutionary analyses were conducted in MEGA7[2].

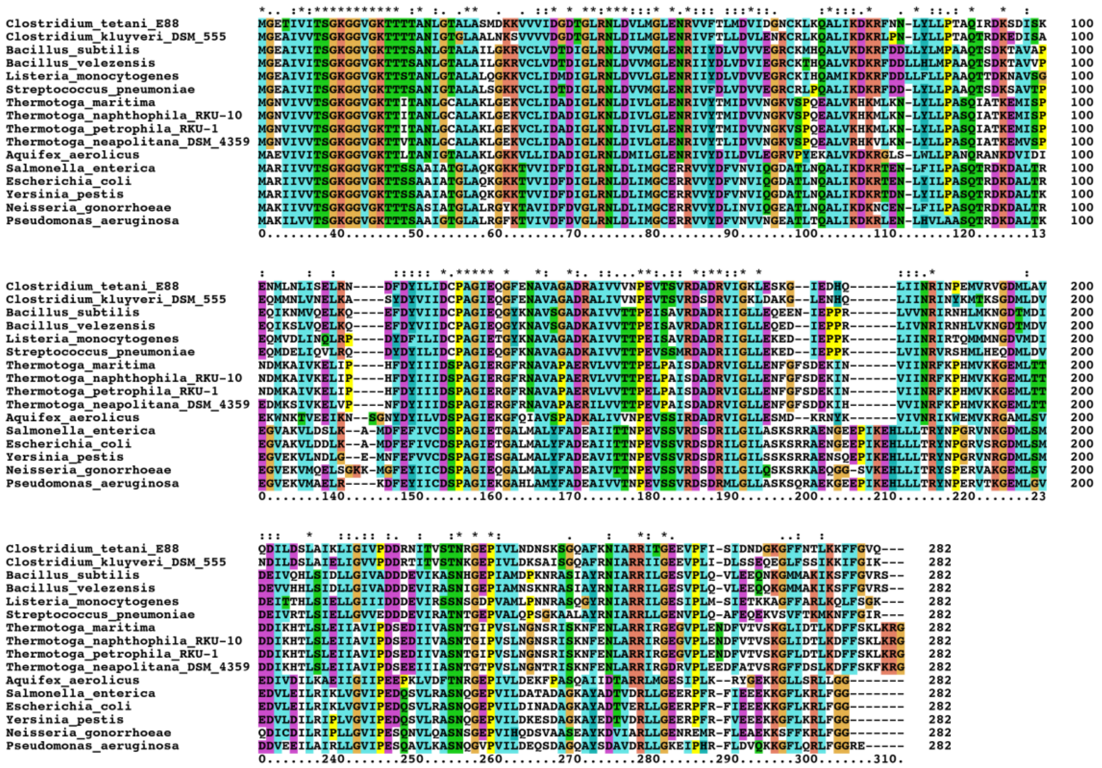

Figure S10. Sequence alignment of MinDs (0-312 aa) from different organisms. MinD in most organisms is conserved. Alignment was performed using the program *ClustalX 2.1*.

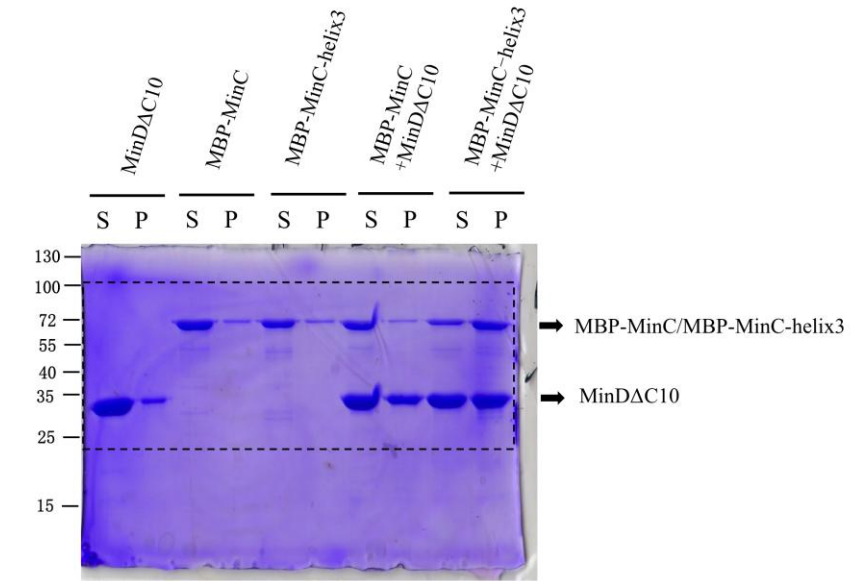

Figure S11. (Related to Fig. 5) Uncropped SDS-PAGE gel related to the co-pelleting experiment. The box in dotted line indicated the cropped gel in Fig. 5.

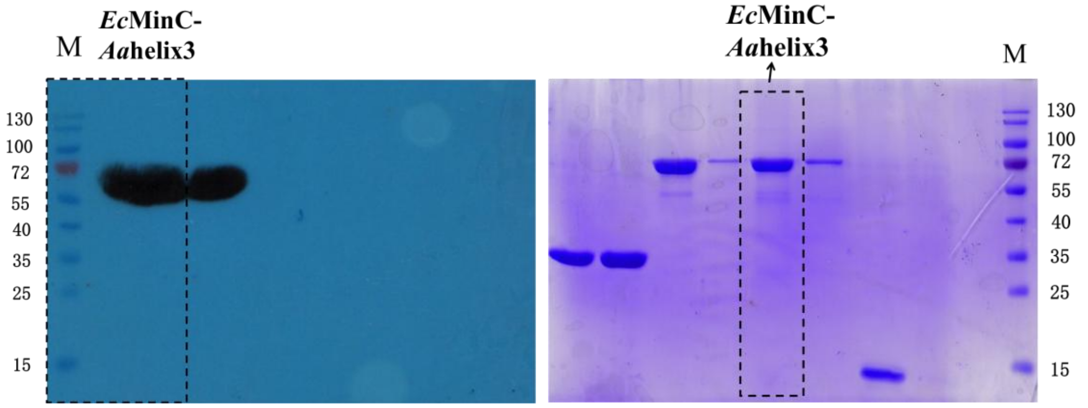

Figure S12. (Related to Fig. S2) Uncropped SDS-PAGE gel and western blot probed with a mouse monoclonal anti-His-HRP conjugated antibody. The box in dotted line indicated the cropped gel in Fig. S2.

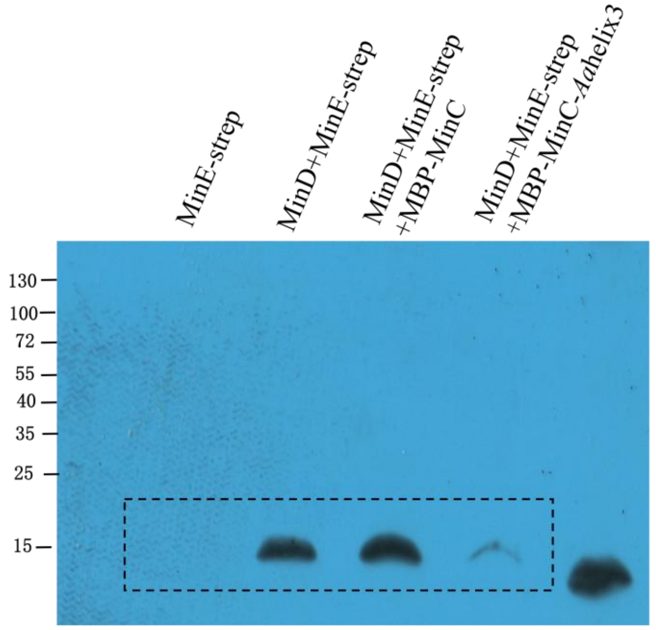

Figure S13. (Related to Fig. S7) Uncropped western blot probed with a mouse monoclonal anti-strep conjugated antibody. The box in dotted line indicated the cropped gel in Fig. S7.

Table S1. Plasmids used in this study

Plasmids	Restriction sites	Fusion tag	Antibiotic resistance
pET-22b-*Ec*MinC	*Nde*I/*Xho*I	C-His	Ampicillin
pET-22b-*Ec*MinC$_{CTD}$	*Nde*I/*Xho*I	C-His	Ampicillin
pET-22b-*Ec*MinC$_{CTD}$-*Aa*helix3	*Nde*I/ /*Xho*I	C-His	Ampicillin
pET-22b-*Ec*MinC	*Nde*I/*Xho*I	C-His	Ampicillin
pET-22b-*Ec*MinC-*Aa*helix3	*Nde*I/ *Xho*I	C-His	Ampicillin
pET-22b-*Ec*MinDΔC10	*Nde*I/*Xho*I	C-His	Ampicillin
pET-28b-MBP-*Ec*MinC	*Nco*I/ *Nde*I/*Xho*I	C-His	Kanamycin
pET-28b-MBP-*Ec*MinC-*Aa*helix3	*Nco*I/ *Nde*I/*Xho*I	C-His	Kanamycin
pET-28b-MBP-*Ec*MinC-T	*Nco*I/ *Nde*I/*Xho*I	No-His	Kanamycin
pET-28b-MBP-*Ec*MinC-*Aa*helix3-T	*Nco*I/ *Nde*I/*Xho*I	No-His	Kanamycin
pET-22b-*Ec*MinE	*Nde*I/*Xho*I	C-strep	Ampicillin

Table S2. Data collection and refinement statistics for *E. coli* MinC$_{CTD}$. Values in parentheses are for the highest resolution shell.

**Crystals**	***E. coli* MinC$_{CTD}$**
Data collection	
Space group	$P\,4_3\,2\,2$
Unit-cell dimensions (Å)	$a = b$ = 65.061, c = 115.926<br>α = β = γ= 90°
Resolution range (Å)	28.98 - 3.00
Observed reflections	66350
Unique reflections	5385
Multiplicity	4
Completeness (%)	99.5
R_{meas} † (%)	9.5
$I/\sigma(I)$	8.6
Refinement statistics	
Resolution (Å)	28.98 - 3.004
R_{work} ‡/R_{free} § (%)	0.2259/0.2776
R.m.s.d bond length (Å)	0.003
R.m.s.d bond angle (°)	0.60
Ramachandran plot, residues in (%)	
Most favored regions	92
Additional allowed regions	7.4
Disallowed regions	0.93
PDB ID	5XDM

† $R_{meas} = \sum_{hkl}\{N(hkl)/[N(hkl)-1]\}^{1/2}\sum_i|I_i(hkl) - \langle I(hkl)\rangle|/\sum_{hkl}\sum_i I_i(hkl)$, where $I_i(hkl)$ is the intensity of the ith observation of reflection hkl and $\langle I(hkl)\rangle$ is the average intensity of reflection hkl.

‡ $R_{work} = \Sigma||F_o| - |F_c||/\Sigma|F_o|$.

§ R_{free} calculated with 10% of all reflections excluded from refinement.

Table S3. Preliminary summary of cell division components from different organisms

Organisms				The C-terminal tail of MinC	MinD	MinE	FtsZ
**Gram-negative**	**Proteobacteria**		*Escherichia coli*	No helix3	Conserved	Yes	Yes
			Pseudomonas aeruginosa	No helix3	Conserved	Yes	Yes
			Neisseria gonorrhoeae	No helix3	Conserved	Yes	Yes
			Salmonella enterica	No helix3	Conserved	Yes	Yes
			Yersinia pestis	No helix3	Conserved	Yes	Yes
	**Thermophile**	**Thermotogae**	*Thermotoga maritima*	C-loop	Conserved	No	Yes
			Thermotoga naphthophila RKU-10	C-loop	Conserved	No	Yes
			Thermotoga neapolitana DSM 4359	C-loop	Conserved	No	Yes
			Thermotoga petrophila RKU-1	C-loop	Conserved	No	Yes
		**Aquificae**	*Aquifex aeolicus*	helix3	Conserved	No	Yes
**Gram-positive**			*Bacillus subtilis*	Probably a helix	Conserved	No (has DivIVA)	Yes
			Bacillus velezensis	Probably a helix	Conserved	No (has DivIVA)	Yes
			Listeria monocytogenes	Probably a helix	Conserved	No (has DivIVA)	Yes
			Streptococcus pneumoniae	Probably a helix	Conserved	No (has DivIVA)	Yes
			Clostridium tetani E88	No helix3	Conserved	Yes	Yes
			Clostridium kluyveri DSM 555	No helix3	Conserved	Yes	Yes
**Archaea**			*Archaeoglobus fulgidus*	No	Conserved	No	Yes
			Methanococcus jannaschii	No	Conserved	No	Yes
			Methanothermobacter thermautotrophicus	No	Conserved	No	Yes

Supplementary References

1. Bohm, G., Muhr, R. & Jaenicke, R. Quantitative analysis of protein far UV circular dichroism spectra by neural networks. *Protein eng.* **5,** 191-5 (1992).

2. Kumar, S., Stecher, G. & Tamura, K. MEGA7: Molecular Evolutionary Genetics Analysis Version 7.0 for Bigger Datasets. *Mol. Biol. Evol.* **33**, 1870-4 (2016).
